# Uric Acid: The Chemistry, Physiology, and Pathology of Uric Acid

**Published:** 1906-08

**Authors:** 


URIC ACID: THE CHEMISTRY, PHYSIOLOGY, AND PATHOLOGY OF URIC ACID, and the Physiologically Important Purin Bodies; with a discussion of the Metabolism in Gout. By Francis H. McCrudden. From Paul B. Hoeber, Medical Books, 69 E. Fifty-ninth St., New York. Paper cover, 308 pages. Price, $2.50, net; canvas, $3, net.

The importance of metabolism in certain diseases, *e. g.*, chronic bone disease, rheumatoid-arthritis, osteo-arthritis, osteo-deformans, etc., is not understood and appreciated as it should be, nor is the role that uric acid plays as a factor of numerous pathological conditions. "Uric acid affects not only the blood, but influences in a similar way the functions, nutrition, and eventually the structure of every organ and tissue of the body, and, as regards infectious disorders has, in some cases, a more important influence than the microbes themselves. As regards the tissues, it controls the production of energy and the production of heat to an extent which, acting as it does, from bone to bone throughout the whole of life, can not but be of enormous importance. But more recent advocates have carried us far beyond this, and we can now say, with absolute certainty, that uric acid controls and conditions the capillary circulation of the whole body, * * * and thus regulates blood-pressure, the heart action, the nutrition of the heart and vessels, the nutrition of the tissue, and all the metabolic phenomena which constitute the life of the body to its minutest cells." (Haig. Uric Acid as a Factor in the Causation of Disease. Sixth edition, 1903.)

Dr. McCrudden has in the book before us treated the subject fully, lucidly and impressively, thereby making a valuable contribution to the literature of the subject and rendering an important service to mankind and the medical profession especially. D.

